# Nonrandom sister chromatid segregation mediates rDNA copy number maintenance in *Drosophila*

**DOI:** 10.1126/sciadv.abo4443

**Published:** 2022-07-27

**Authors:** George J. Watase, Jonathan O. Nelson, Yukiko M. Yamashita

**Affiliations:** ^1^ Whitehead Institute for Biomedical Research, Department of Biology, Massachusetts Institute of Technology, Cambridge, MA 02142, USA.; ^2^ Howard Hughes Medical Institute, 455 Main Street, Cambridge, MA 02142, USA.

## Abstract

Although considered to be exact copies of each other, sister chromatids can segregate nonrandomly in some cases. For example, sister chromatids of the X and Y chromosomes segregate nonrandomly during asymmetric division of male germline stem cells (GSCs) in *Drosophila melanogaster*. Here, we demonstrate that the ribosomal DNA (rDNA) loci, which are located on the X and Y chromosomes, and an rDNA binding protein Indra are required for nonrandom sister chromatid segregation (NRSS). We provide the evidence that NRSS, following unequal sister chromatid exchange, is a mechanism by which GSCs recover rDNA copy number, counteracting the spontaneous copy number loss that occurs during aging. Our study reveals an unexpected role for NRSS in maintaining germline immortality through maintenance of a vulnerable genomic element, rDNA.

## Teaser

rDNA copy number maintenance by nonrandom sister chromatid segregation contributes to germline immortality in *Drosophila*.

## INTRODUCTION

Sister chromatids, generated through the precise process of DNA replication, are considered identical. Nevertheless, it has been proposed that sister chromatids might carry distinct information or mutation loads, and their nonrandom segregation may underlie asymmetric cell division (*1*–*3*). One of these hypotheses is called the immortal strand hypothesis, wherein the DNA strand that contains the older template is inherited by stem cells to avoid the accumulation of replication-induced mutations (*2*, *4*). Alternatively, the difference in epigenetic information between two sister chromatids was proposed to govern and/or be carried by nonrandom sister chromatid segregation (NRSS) (*5*, *6*). In these cases, the differences between sister chromatids were assumed to be distributed along the entire chromosomes. However, the lack of understanding on the cis-elements and the associated molecular mechanisms that govern NRSS has prevented the investigation of the physiological relevance of NRSS.

**Fig. 1.**
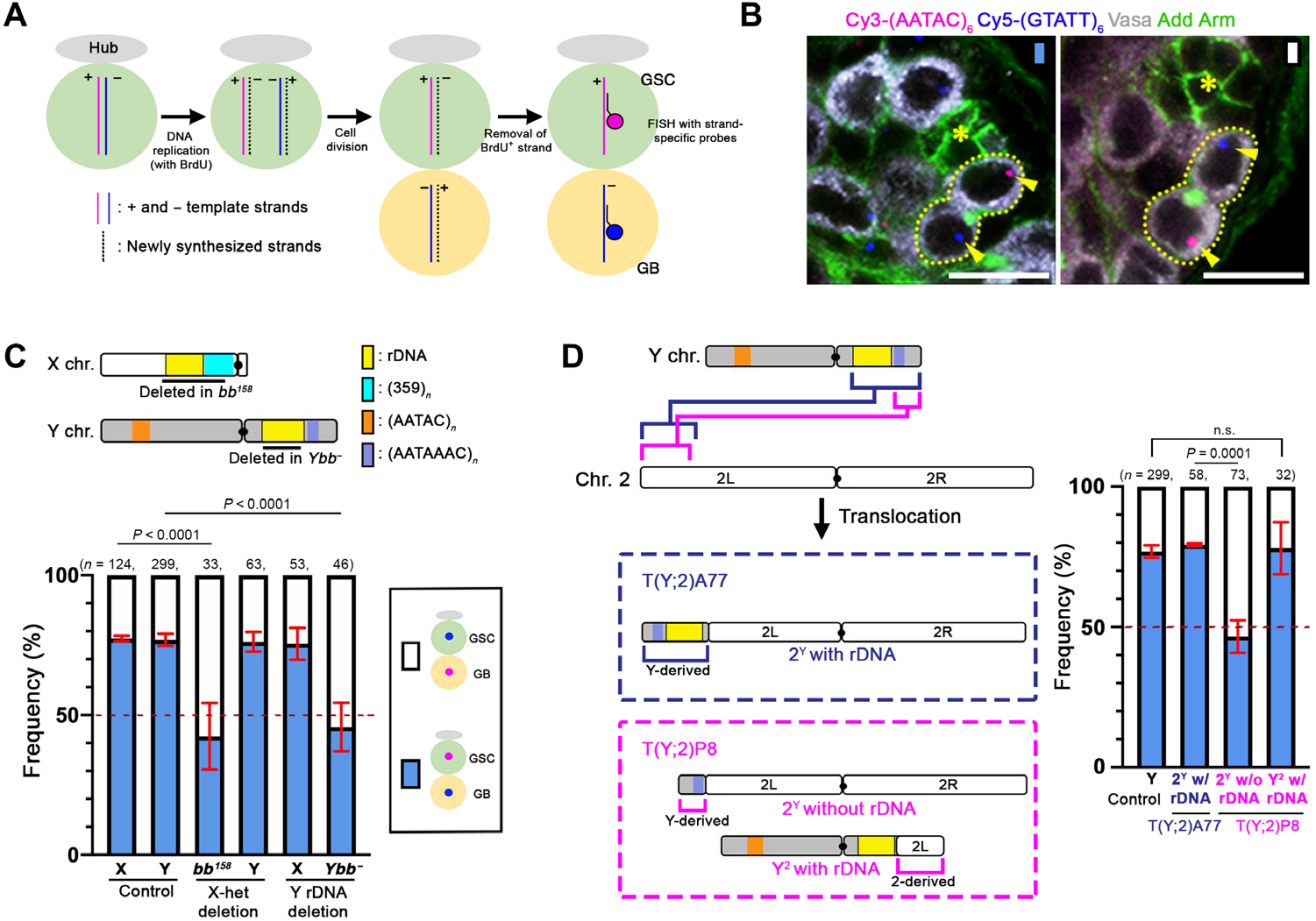
rDNA loci are required for NRSS of the X and Y chromosomes in *D. melanogaster.* (**A**) CO-FISH to assess NRSS. Plus (+) versus minus (−) templated strands are indicated by pink and blue lines, and newly synthesized strands are indicated by black dotted lines. Following removal of 5-bromodeoxyuridine (BrdU) containing newly synthesized strands, strand-specific probes were applied to distinguish pink versus blue templated strands. (**B**) Representative images of Y chromosome CO-FISH results where a GSC inherits the “pink” strand [(AATAC)*_n_*], whereas a GB inherits the “blue” strand [(GTATT)*_n_*] (left) and the opposite pattern (right). The hub, the stem cell niche to which GSCs are attached, is indicated by an asterisk; GSC-GB pairs are outlined by dotted lines; and the CO-FISH signals are indicated by arrowheads. Vasa, germ cells; arm, hub; add, the connection between GSC and GB. Scale bars, 10 μm. (**C** and **D**) Schematics of *D. melanogaster* X and Y chromosomes (C) and Y-2 translocation chromosomes (D). Summary of sister chromatid segregation patterns in indicated genotypes is shown (see tables S1 and S2). Data are shown as means ± SD from three independent experiments. *n*, number of GSC-GB pairs scored; *P* values, Fisher’s exact test. n.s., not significant.

Using *Drosophila melanogaster* male germline stem cells (GSCs) as a model system, where asymmetric stem cell division can be observed at a single-cell resolution, we previously showed that the X and Y chromosomes exhibit notably biased sister chromatid segregation (*7*). By using chromosome orientation fluorescence in situ hybridization (CO-FISH) with chromosome-specific probes (Fig. 1A), (+)-strand templated versus (−)-strand templated sister chromatids of each chromosome can be distinguished (Fig. 1, A and B). If sister chromatids are equivalent, then (+)- versus (−)-strand templated sister chromatids would segregate to the GSC or GB (gonialblast; the differentiating daughter of a GSC) at random (50:50). Although we observed random sister chromatid segregation for autosomes (chromosomes 2 and 3), the X and Y chromosomes segregated their sister chromatids nonrandomly, with a specific strand segregating to GSC in ~80% of observed divisions (Fig. 1C, “pink strand”) (*7*). This demonstrated that sister chromatids, which supposedly carry the same genetic information, can be distinguished and segregated nonrandomly during asymmetric stem cell division. However, the underlying mechanism remained elusive.

In this study, we show that ribosomal DNA (rDNA) loci are required for NRSS. We further identify the rDNA binding protein Indra, which we show is required for NRSS and rDNA copy number maintenance. This is the first study to identify chromosomal elements that are required for NRSS. We further present the results that collectively suggest that NRSS is the critical process to maintain rDNA copy number, which is inherently unstable because of its tandemly repeated structure. We propose that transgenerational maintenance of rDNA copy number, mediated by NRSS, is the key to sustaining germline immortality.

## RESULTS

### rDNA loci are responsible for NRSS

To elucidate the underlying molecular mechanism of NRSS, we sought to identify the chromosomal loci that mediate NRSS and found that rDNA is required. By using various deletion lines, we found that an X chromosome without rDNA, *Df(1)bb^158^* (*bb^158^* hereafter), as well as a Y chromosome without rDNA (*Ybb^−^*), exhibited randomized sister chromatid segregation (Fig. 1C and table S1). The intact Y chromosome in the GSCs that carry *bb^158^* chromosome still exhibited NRSS. Likewise, the intact X chromosome in the GSCs that carry *Ybb^−^* chromosome still exhibited NRSS (Fig. 1C and table S1). Moreover, a chromosome 2 containing an rDNA locus translocated from the Y chromosome also exhibited NRSS [“2^Y^ with rDNA” in T(Y;2)A77 translocation; Fig. 1D and table S2], suggesting that rDNA is sufficient to induce NRSS. As a critical control, a chromosome 2 carrying a similar translocation from the Y chromosome that does not include the rDNA did not exhibit NRSS [“2^Y^ without rDNA” in T(Y;2)P8 translocation; Fig. 1D and table S2]. No elements other than rDNA locus are commonly involved in chromosomes used here: X chromosome without rDNA (*bb^158^*), Y chromosome without rDNA (*Ybb^−^*), and chromosome 2 with rDNA translocated from Y chromosome [T(Y;2)A77], strongly suggesting that rDNA is the element responsible for NRSS. These results together suggest that the rDNA loci likely act as cis-elements to mediate NRSS, without influencing the NRSS of other chromosomes in trans. This is the first demonstration that a specific region of a chromosome is responsible for NRSS, opposing the widely held speculation that NRSS depends on chromosome-wide information such as epigenetic information and replication-induced mutations (*8*).

### rDNA binding protein Indra is required for NRSS

**Fig. 2.**
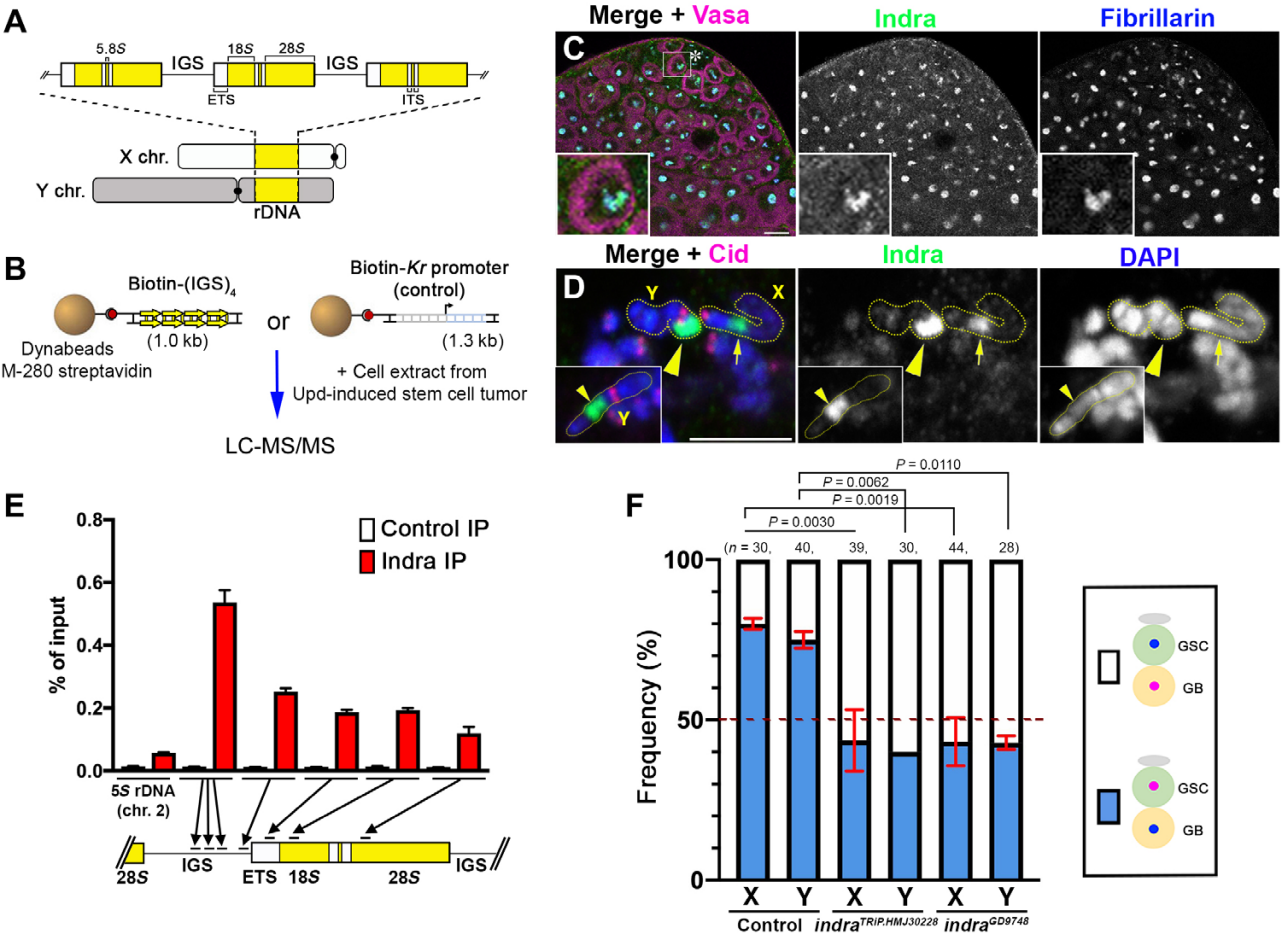
Indra is a previously uncharacterized zinc finger protein that binds to rDNA and mediates NRSS. (**A**) Schematic of rDNA loci in *D. melanogaster*. (**B**) Experimental scheme to isolate IGS-binding proteins (see Materials and Methods). Upd, unpaired. (**C**) Localization of Indra at the apical tip of the testis. The hub is indicated by an asterisk. An enlarged image of a GSC is shown in the inset. Fibrillarin, nucleolus; vasa, germ cells. Scale bar, 10 μm. (**D**) Localization of Indra on a metaphase chromosome spread from germ cells. The X rDNA locus (arrow) and Y rDNA locus (arrowhead) can be identified by their relative location to the centromere (Cid), according to well-established cytological location of rDNA in *Drosophila*. An additional example of the Y chromosome is shown in the inset. Scale bar, 5 μm. (**E**) Indra ChIP-qPCR showing enrichment of Indra on rDNA/IGS. The 5*S* rDNA sequence on chromosome 2 outside of the rDNA loci was used as a negative control. Mean and SD from three technical replicates of qPCR are shown. Similar results were obtained from two biological replicates. (**F**) Summary of sister chromatid segregation patterns upon knockdown of *indra* (see table S4). Data are shown as means ± SD from three independent experiments. *n*, number of GSC-GB pairs scored; *P* values, Fisher’s exact test.

To begin to understand how rDNA mediates NRSS, we sought to isolate rDNA binding proteins from the GSC extract. Each rDNA locus consists of ~150 to 225 repeated rDNA units to support the high demand of ribosome biogenesis (*9*). Each rDNA unit contains the 18*S*, 5.8*S*/2*S*, and 28*S* rRNA genes and three spacer sequences [the external transcribed spacer (ETS), internal transcribed spacer (ITS), and intergenic spacer (IGS)] (Fig. 2A). The Y chromosome of *Drosophila simulans*, a species closely related to *D. melanogaster*, has IGS repeats but no rRNA genes, ETS, or ITS (*10*) yet exhibited NRSS (fig. S1 and table S1). We hypothesized that IGS may be responsible for NRSS. Accordingly, we isolated IGS-binding proteins by mass spectrometry (MS), followed by a secondary screen based on subcellular localization (Fig. 2B and table S3). In this study, we focus on a previously uncharacterized zinc finger protein, CG2199, which we named Indra after the Hindu god who lost immortality due to a curse from Durvasa. Using a specific anti-Indra antibody (fig. S2A) and an Indra–green fluorescent protein (GFP) line, we found that Indra localizes to the nucleolus (the site of rDNA transcription) in interphase (Fig. 2C and fig. S3A) and rDNA loci during metaphase (Fig. 2D and fig. S3B). Chromatin immunoprecipitation quantitative polymerase chain reaction (ChIP-qPCR) further demonstrated that Indra preferentially binds to IGS (Fig. 2E).

Notably, RNA interference (RNAi)–mediated knockdown of *indra* in the germ line (fig. S2A) compromised NRSS for both the X and Y chromosomes (Fig. 2F and table S4). Although our original reasoning of IGS being responsible for NRSS was hypothetical (based on logical deduction), the fact that the IGS-binding protein Indra is required for NRSS supports that IGS is a critical player of NRSS. However, insertion of eight copies of IGS to X chromosome without rDNA (*bb^158^*) did not restore NRSS (fig. S4): Considering that rDNA loci contain thousands of IGS repeats, it is possible that a much higher number of repeats are needed to confer NRSS. Alternatively, IGS may need to be placed in the context of rDNA locus to mediate NRSS (e.g., transcription and proximity to other rDNA elements). Together, these results suggest that IGS and its binding protein Indra are important for NRSS.

### Indra is required for rDNA copy number maintenance

**Fig. 3.**
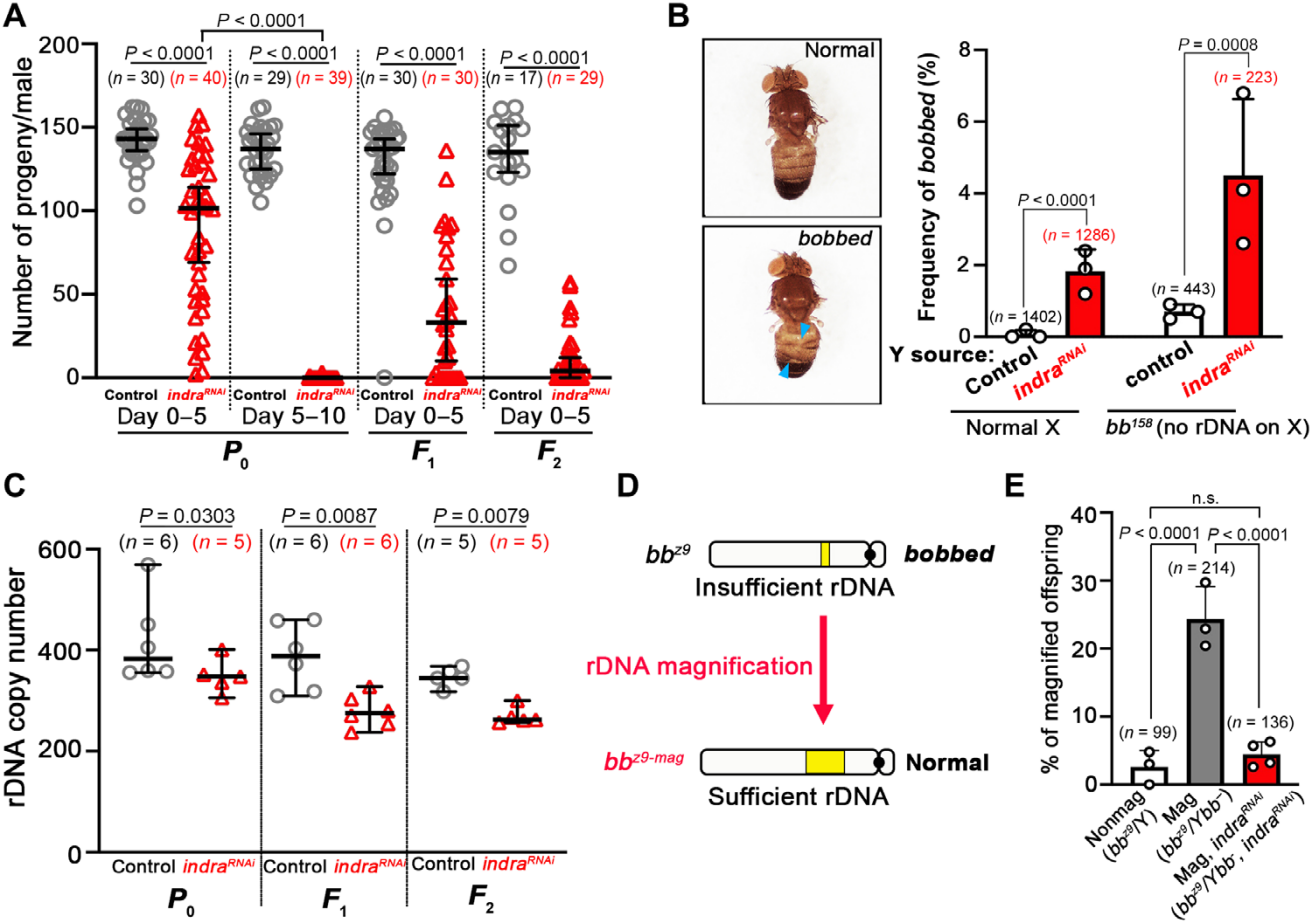
*indra* is required for rDNA copy number maintenance. (**A**) Fertility of control and *nos-gal4*>*UAS-indra^TRiP.HMJ30228^* males across generations (*P*_0_, *F*_1_, and *F*_2_). Total numbers of progeny from 0- to 5-day-old males in each generation and 5- to 10-day-old males in *P*_0_ were scored. Data are shown as median with 95% confidence interval and individual data points. *n*, number of individual crosses scored; *P* value, two-tailed Mann-Whitney test. (**B**) Frequency of *bobbed* animals in progeny of 0- to 5-day-old control and *nos-gal4>UAS-indra^TRiP.HMJ30228^* males. Mean and SD from three independent experiments with individual data points are shown. *n*, total number of progeny scored; *P* values, two-tailed chi-squared test. Examples of normal and *bobbed* cuticle phenotypes are shown on the left. (**C**) 28*S* rRNA gene copy number in the testes from 0- to 5-day-old control and *nos-gal4*>*UAS-indra^TRiP.HMJ30228^* males in successive generations (*P*_0_, *F*_1_, and *F*_2_) assessed by ddPCR. Data are shown as median with 95% confidence interval and individual data points. *n*, number of individual crosses scored; *P* value, two-tailed Mann-Whitney test. (**D**) Schematic diagram of rDNA magnification assay (see fig. S5 for the details). Magnification was detected by normal cuticle phenotype in the offspring. (**E**) Frequency of rDNA magnification under the indicated genotypes/conditions. Data shown as mean and SD from four (*nos-gal4*>*UAS-indra^GD9748^* and *UAS-Dcr-2*) or three (the rest) independent experiments with individual data points. *n*, total number of progeny scored; *P* values, Fisher’s exact test.

To understand the function of Indra, we examined phenotypic outcome of *indra* depletion. RNAi-mediated depletion of *indra* (*nos-gal4>UAS-indra^TRiP.HMJ30228^*) resulted in markedly fewer progeny compared to control (Fig. 3A, *P*_0_). Some of the offspring from *indra^TRiP.HMJ30228^* males exhibited a *bobbed* phenotype, a hallmark of rDNA copy number insufficiency characterized by abnormal cuticle patterns on the abdomen (Fig. 3B) (*11*). The frequency of *bobbed* flies increased when the Y chromosome from *indra^TRiP.HMJ30228^* fathers was placed in the background of *bb^158^*, the X chromosome that lacks rDNA (Fig. 3B). Quantitative droplet digital PCR (ddPCR) confirmed that rDNA copy number was reduced in *indra^TRiP.HMJ30228^* animals (Fig. 3C, *P*_0_). Depletion of *indra* over successive generations resulted in a progressive loss of fecundity (Fig. 3A, *F*_1_ and *F*_2_) associated with a reduction in rDNA copy number (Fig. 3C, *F*_1_ and *F*_2_). These results suggest that *indra* is required for maintaining fecundity over generations through the maintenance of rDNA copy number.

Moreover, we found that *indra* is required for “rDNA magnification,” a phenomenon by which an X chromosome with insufficient rDNA copy number is induced to recover copy number (Fig. 3, D and E, and fig. S5) (*12*, *13*). An X chromosome with insufficient rDNA copy number (*bb^z9^*) normally stably maintains this low copy number, but when combined with Y chromosome that entirely lacks rDNA (*Ybb^−^*), X rDNA locus (*bb^z9^*) is induced to increase the rDNA copy number, leading to rDNA magnification (Fig. 3D and fig. S5). Whereas *bb^z9^/Ybb^−^* males have *bobbed* phenotype due to rDNA insufficiency, some of their offspring exhibit wild-type cuticle as a result of rDNA magnification in the germ line of *bb^z9^/Ybb^−^* males. rDNA magnification is not induced if *bb^z9^* is paired with the normal Y chromosome with sufficient amount of rDNA (*bb^z9^*/Y). Under a magnifying condition (*bb^z9^/Ybb^−^*), ~25% of the offspring exhibited rDNA magnification, whereas *indra* depletion blocked the magnification (*bb^z9^/Ybb^−^*, *nos-gal4*>*UAS-indra^GD9748^*, and *UAS-Dcr-2*) (Fig. 3E), suggesting that *indra* is required for rDNA magnification. These results suggest that *indra* contributes to rDNA copy number maintenance by promoting rDNA copy number recovery.

### rDNA undergoes unequal sister chromatid exchange during rDNA magnification

**Fig. 4.**
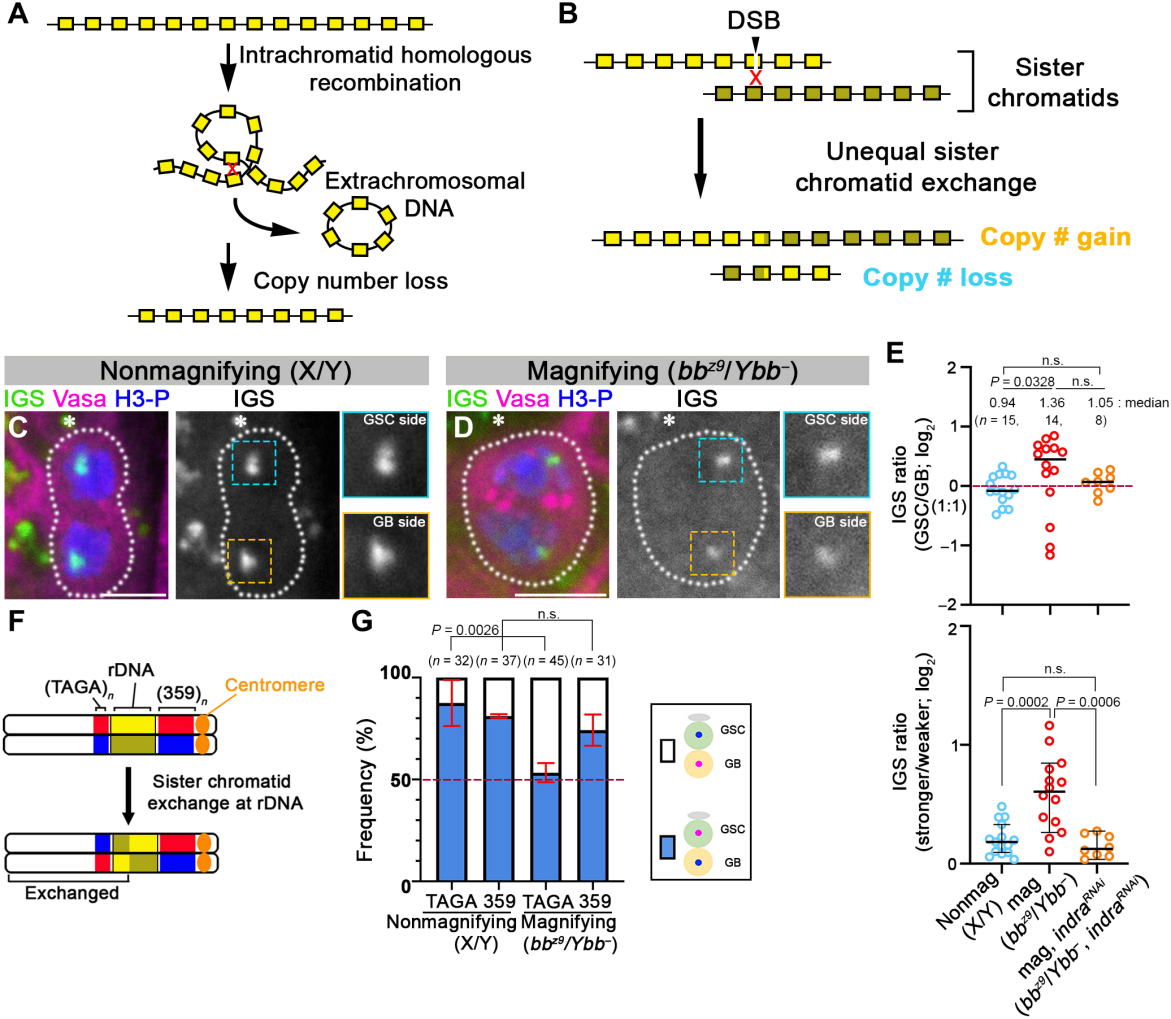
rDNA loci undergo sister chromatid exchange and segregate asymmetrically in GSCs during rDNA magnification. (**A**) Diagram of spontaneous rDNA copy number loss by intrachromatid recombination. (**B**) A proposed model for rDNA copy number recovery by USCE. DSB, double-strand break. (**C** and **D**) DNA FISH for IGS during anaphase in GSCs under normal (*yw*) (C) and magnifying (*bb^z9^/Ybb^−^*) (D) conditions. The hub is indicated by an asterisk. An enlarged image of the IGS signal from GSC and GB sides is shown in the inset. Scale bars, 5 μm. (**E**) Quantification of IGS signal intensity presented as GSC side/GB side (top) or stronger/weaker (bottom) during anaphase in GSCs in control and *nos-gal4*>*UAS-indra^GD9748^* and *UAS-Dcr-2* males. The median and individual data points are shown. A 95% confidence interval is also shown in the bottom panel. *n*, number of anaphase cells scored; *P* value, two-tailed Mann-Whitney test. Note that because of rare cases where the GB side exhibited stronger IGS signal than GSC side, the data did not reach statistical significance between control and *indra^GD9748^* under magnifying conditions in the top panel. (**F**) Diagram of the X chromosome showing the location of the rDNA and the 359-bp and (TAGA)*_n_* repeats. Sister chromatid exchange at rDNA loci would flip (TAGA)*_n_* segregation pattern relative to 359 bp. (**G**) Summary of sister chromatid segregation patterns assessed by 359-bp and (TAGA)*_n_* probes under the indicated genotypes/conditions (see table S5). Data are shown as means ± SD from three independent experiments. *n*, number of GSC-GB pairs scored; *P* values, Fisher’s exact test.

Although the repetitiveness of rDNA loci is critical to support ribosome biogenesis, it also makes rDNA loci susceptible to intrachromatid recombination, which leads to spontaneous copy number loss (Fig. 4A). To maintain the integrity of rDNA loci, copy number loss must be counteracted by copy number recovery. In yeast, rDNA copy number recovery is mediated by unequal sister chromatid recombination (*14*). In *Drosophila*, extensive studies have led to the hypothesis that rDNA magnification uses unequal sister chromatid exchange (USCE) (*9*, *12*, *13*, *15*, *16*). Previously, we proposed that rDNA magnification, which was originally discovered with mutant chromosomes with extremely low rDNA copy number, is a mechanism that operates in wild-type flies to maintain rDNA copy number through generations, counteracting spontaneous copy number loss during aging (*17*). USCE allows for copy number recovery on one of the sister chromatids at the expense of the other (Fig. 4B) (*12*), generating asymmetry in rDNA copy number between two sister chromatids (Fig. 4B). We hypothesized that this asymmetry may underlie NRSS. Such a hypothesis would explain why *indra* regulates NRSS and rDNA magnification.

Notably, using DNA FISH, asymmetry in rDNA amount was detected during anaphase in GSCs under “magnifying conditions” (*bb^z9^/Ybb^−^*), where the GSCs preferentially inherited the stronger signal (Fig. 4, D and E). As an important control, asymmetry in rDNA amount was not observed in flies with sufficient rDNA copy number (Fig. 4, C and E). The difference in the rDNA signal between GSC and GB (GSC signal divided by GB signal) was statistically significant (Fig. 4E, top). These results suggest that asymmetry in rDNA copy number is created between two sister chromatids specifically under magnifying conditions, supporting the notion that USCE is induced only during rDNA magnification (Fig. 4B). This asymmetry in rDNA amount between two sister chromatids was lost upon *indra* depletion (*bb^z9^/Ybb^−^*, *nos-gal4>UAS-indra^GD9748^*, and *UAS-Dcr-2*) (Fig. 4E, top). Note that the asymmetry score in the rDNA amount (GSC/GB) did not reach statistical significance between control and *indra^GD9748^* under magnifying conditions (Fig. 4E, top); however, plotting the ratio of stronger over weaker rDNA signal (Fig. 4E, bottom), it became clear that the magnifying condition induced copy number asymmetry in control (*bb^z9^/Ybb^−^*), whereas this asymmetry itself was abrogated following depletion of *indra* (*bb^z9^/Ybb^−^*, *nos-gal4>UAS-indra^GD9748^*, and *UAS-Dcr-2*) (Fig. 4E, bottom). These results show that *indra* may be involved not only in NRSS but also in generating copy number asymmetry through USCE.

These results are consistent with a model in which rDNA magnification is mediated by USCE, and the sister chromatid with increased rDNA copy number is selectively retained by GSCs via NRSS. We further tested this idea by CO-FISH with additional probes. The probe for the 359–base pair (bp) repeat, located proximal to the rDNA (Fig. 4F), was used to detect NRSS in the experiments described above (Fig. 1). (TAGA)*_n_*, which is located distal to the rDNA, also exhibited NRSS under control (nonmagnifying) conditions (Fig. 4G and table S5), demonstrating that the entire rDNA locus and the flanking regions exhibit NRSS. Notably, under magnifying conditions, whereas 359-bp maintained NRSS, (TAGA)*_n_* exhibited random segregation, suggesting that sister chromatid exchange occurred between 359-bp and (TAGA)*_n_*, most likely within the rDNA locus (Fig. 4F). Together, these results suggest that rDNA magnification is associated with USCE, followed by the retention of the sister chromatids with increased rDNA copy number by GSCs. These results also identify GSCs as the most likely cell population that undergoes rDNA magnification.

## DISCUSSION

In this study, we provide the first evidence that NRSS is mediated by a localized chromosomal element, as opposed to a previously held assumption/proposal that NRSS carries epigenetic information or replication-induced mutations that are present along the chromosome (*8*). By identifying rDNA and its binding protein Indra as an element/factor responsible for NRSS, we provide the first mechanistic framework of how NRSS may occur. We further propose that NRSS plays an important role in maintaining the rDNA copy number through generations, counteracting spontaneous copy number loss that occurs during repeated cell divisions. Although our work does not eliminate the possibility of the presence of other forms of NRSS, such as governed by the information that is present across the entire chromosome as speculated previously, our work provides a new framework in investigating the mechanism and implication of NRSS.

In *Drosophila*, the body of work (*9*, *12*, *13*, *16*, *18*) has established the phenomenon of rDNA magnification, where rDNA copy number can increase likely via USCE, but it remained unknown in which cell type this may occur and for what purpose (*9*). Our study suggests that rDNA magnification is the mechanism that is essential to maintain germline immortality, as continuous loss of rDNA copy number in successive generations is expected to cause extinction of the lineage. Moreover, our study indicates that the rDNA magnification likely occurs in GSCs through biased segregation of the sister chromatids, allowing GSCs to inherit more rDNA copy number following USCE. Although other cell types [e.g., spermatogonia (SG)] may undergo USCE to gain the copy number, GSCs have a critical advantage over SGs in rDNA magnification due to its ability to repeatedly inherit the sister chromatid that gained the copy number. This may explain why rDNA magnification can increase more than twice in one generation of an animal, the observation that was deemed incompatible with USCE model (*19*), because a single USCE event would magnify up to two times. However, GSCs would be able to magnify multiple times over multiple cell divisions, leading to more than twice expansion of rDNA copy number within one generation of an animal. The fate of GBs that lost the rDNA copy number upon USCE remains elusive. We speculate that GBs may be eliminated via cell death if the copy number loss is too large, whereas they may produce functional sperm if they still retain sufficient rDNA copy number. Note that GSCs produce many GBs via repeated divisions, whereas individual GBs produce only 64 sperms (through four mitotic divisions and two meiotic divisions). Therefore, the impact of GBs inheriting the decreased rDNA copy number would be minimal at an organismal level.

The underlying mechanisms that allow GSCs to inherit the sister chromatid that gained rDNA copy number remain elusive. However, the results presented in this study provide a few critical insights. Under magnifying conditions, where USCE occurs, it is the (TAGA)*_n_* repeats whose segregation pattern is randomized, whereas 359-bp repeats maintain NRSS. This suggests that the 359-bp side of the rDNA is responsible for the retention in GSCs. This side of the chromosome contains the centromere, whose asymmetry has been suggested to mediate nonrandom segregation of chromosomes (*20*, *21*). However, we have no evidence thus far to suggest that the centromere is responsible for NRSS of the X and Y chromosomes. In addition, *bb^158^* chromosome, which does not have any rDNA but retains most of 359-bp and the entire centromere, compromises NRSS (Fig. 1C). Therefore, it is highly unlikely that 359-bp or the centromere contains sufficient information to mediate NRSS.

Another important insight comes from the sister chromatid segregation pattern under the nonmagnifying condition. Under nonmagnifying conditions, USCE appears to be rare, based on the equal amount of IGS FISH signal in anaphase GSCs (Fig. 4, C and E) and based on the fact that both 359-bp repeats and (TAGA)*_n_* repeats segregate nonrandomly (Fig. 4G). This means that GSCs still faithfully retain a specific strand (red strand) even without asymmetry in rDNA copy number (Fig. 4, F and G). This suggests that NRSS is not mediated by actual copy number differences but rather implies that sister chromatids (of rDNA loci) may have additional inherent asymmetries. Currently, we can only speculate what might be such an asymmetry: Such an asymmetry may correlate with the propensity of a specific sister chromatid to gain rDNA copy number, and should USCE occur. An attractive candidate for such an asymmetry is the molecular asymmetry during DNA replication that is specific to rDNA loci. It is well established that DNA replication occurs unidirectionally in rDNA loci (*14*, *22*) due to the presence of a replication fork block on one side of the replication origin (fig. S6A). Accordingly, one sister chromatid is mostly replicated as the leading strand, whereas the other is mostly replicated as the lagging strand. In yeast, the DNA break that induces rDNA copy number recovery is known to occur on the leading strand (the strand mostly replicated as the lagging strand) at the replication fork block (fig. S6B) (*23*). If this is universal, then the broken end of the leading strand has limited choices as to where to recombine with the sister chromatid to repair the DNA break. The broken end would not recombine with a region that is not yet replicated. The recently replicated region of the lagging strand, where Okazaki fragments have not been processed, may not be a good substrate for sister chromatid recombination either. The remaining possible region would be the sister chromatid that was replicated as the leading strand (fig. S6B). If this happens, then the strand mostly replicated as the lagging strand is likely to gain the copy number. Thus, we speculate that the mechanism that mediates NRSS may have the ability to distinguish leading versus lagging strands and specifically connects the lagging strand to the GSC side.

Note that the *Drosophila* male GSCs have been shown to exhibit asymmetric segregation of the old versus new histones (histones H3 and H4), where old histones were observed to be more concentrated on the GSC side (*24*–*27*). It remains unclear how the histones’ nonrandom segregation between GSCs and GBs is related to NRSS described in this study. Considering the fact that the autosomes do not exhibit NRSS (*7*), it is unlikely that NRSS is directly linked to the asymmetric segregation of old versus new histones, which is deemed to occur at all chromosomes. It awaits future investigation how the NRSS and histone inheritances may be mechanistically and biologically linked with each other.

Our study reveals the unexpected molecular mechanisms and biological significance of NRSS. We propose that NRSS is a key process to recover and maintain inherently unstable rDNA copy numbers, such that the integrity of the germline genome is upheld over generations, supporting germline immortality. Future work is required to understand how rDNA copy number differences between sister chromatids are recognized and faithfully segregated to the GSCs to achieve rDNA copy number recovery.

## MATERIALS AND METHODS

### Fly husbandry and strains

All fly stocks were raised on standard Bloomington medium at 25°C containing 0.15% of Tegosept as antifungal (no propionic acid was added). The following fly stocks were used: bb^158^, y^1^/Dp(1;Y)y^+^/C(1)*; ca^1^ awd^K^ (BDSC3143); FM6/C(1)DX, y* f^1^/Y (BDSC784); *UAS-indra^TRiP.HMJ30228^* (BDSC63661); *UAS-Dcr-2* (BDSC24650); and *indra-GFP* (BDSC67660; http://flybase.org/reports/FBti0186577) were obtained from the Bloomington Drosophila Stock Center. *y^1^ eq^1^/Df(YS)bb^−^* (DGRC101260); *T(Y;2)A77, B^S^, y^+^/SM1; C(1)RM, y^1^/C(1;Y)1, y^1^* (DGRC130079); and *T(Y;2)P8, B^S^, y^+^/SM1; C(1)RM, y^1^/C(1;Y)1, y^1^* (DGRC130170) were obtained from the Kyoto Stock Center. *D. simulans W^501^*(DSSC14021-0251.195) was obtained from the National Drosophila Species Stock Center. *UAS-indra^GD9748^* (v20839) was obtained from the Vienna Drosophila Resource Center. *nos-gal4* (*28*), *UAS-Upd* (*29*), *tub-gal80^ts^* (*30*), and *nos-gal4* without VP16 (*31*) have been previously described. The fly that inserted eight copies of 240-bp IGS to X chromosome was generated by phiC31 site–directed integration into the *Drosophila* genome. The 240-bp IGS sequence in *D. melanogaster* (*32*) was synthesized by gene synthesis service from Thermo Fisher Scientific (GeneArt Gene Synthesis) and was cloned into pattB vector to insert specific integration site on X chromosome (attP18). All injection and selection of flies containing integrated transgene were performed by BestGene Inc. (Chino Hills, CA). The bb^158^ background–inserted animals were obtained by recombination between X chromosome with IGS insertion and bb^158^ chromosome.

To examine the sister chromatid segregation patterns of the 2^Y^ and Y^2^ chromosomes, *T(Y;2)A77/SM1; C(1)RM/O* or *T(Y;2)P8/SM1; C(1)RM/O* females were crossed to *yw* males, and the resulting *T(Y;2)A77/+; X/O* and *T(Y;2)P8/+; X/O* male flies were examined. The details of the translocation are shown in Fig. 1D.

Two independent RNAi lines, *UAS-indra^TRiP.HMJ30228^* and *UAS-indra^GD9748^*, were used to knock down *indra* specifically in early germ cells using *nos-gal4* as the driver. *UAS-indra^GD9748^* was combined with *UAS-Dcr-2* to increase RNAi efficiency. The knockdown efficiency of these RNAi lines was validated by immunostaining using an anti-Indra antibody (fig. S2A). Since *nos-gal4>UAS-indra^TRiP.HMJ30228^* results in severe germ cell loss due to high RNAi efficiency (Fig. 3A and fig. S2A), a temperature-sensitive GAL4 inhibition system (*tub-gal80^ts^*; *nos-gal4*Δ*VP16>UAS-indra^TRiP.HMJ30228^*) was used as necessary (e.g., Fig. 2F). Upon shifting from the permissive temperature (18°C) to the nonpermissive temperature (29°C), GSCs were lost gradually over 2 to 4 days (fig. S2, B and C), and the CO-FISH assay (Fig. 2F) was conducted 3 days after temperature shift. In assays that required a sustained germ line (e.g., magnification assays; Figs. 3E and 4E), we used *nos-gal4>UAS-indra^GD9748^* and *UAS-Dcr-2*.

### Immunofluorescence staining and confocal microscopy

*Drosophila* adult testes were dissected in phosphate-buffered saline (PBS), transferred to 4% formaldehyde in PBS, and fixed for 30 min. The testes were then washed in PBST (PBS containing 0.1% Triton X-100) for at least 30 min, followed by incubation with primary antibody in 3% bovine serum albumin (BSA) in PBST at 4°C overnight. Samples were washed for 60 min (3× washes for 20 min) in PBST, incubated with secondary antibody in 3% BSA in PBST at 4°C overnight, washed as above, and mounted in VECTASHIELD with 4′,6-diamidino-2-phenylindole (DAPI; Vector Laboratories, Burlingame, CA). To examine Indra localization on mitotic chromosome spreads, *Drosophila* third instar larval testes were dissected in PBS, transferred to 0.5% sodium citrate, incubated for 10 min, fixed in 4% formaldehyde in PBS for 4 min, and then squashed between the cover slip and slide glass. The sample was frozen in liquid nitrogen, the cover slip was removed and immediately washed in PBS, followed by immunofluorescence staining as described above, except that the incubation was performed on the slide glass in a humid chamber with the sample covered with a small piece of parafilm.

The primary antibodies used were as follows: rabbit anti-vasa (1:200; d-26, Santa Cruz Biotechnology, Santa Cruz, CA), mouse anti-adducin–like [1:20; 1B1, developed by H. D. Lipshitz, obtained from Developmental Studies Hybridoma Bank (DSHB)] (*33*), mouse anti-armadillo (1:100; N2 7A1, developed by E. Wieschaus, obtained from DSHB) (*34*), rat anti-vasa (1:20; developed by A. C. Spradling and D. Williams, obtained from DSHB), mouse anti-fibrillarin (1:200; 38F3, Abcam), and chicken anti-Cid (1:500) (*35*). The anti-Indra antibody was generated by injecting a peptide (RKITDVLETITHRSIPSSLPIKIC) into guinea pig (Covance, Denver, PA) and used at a dilution of 1:500. Specificity of the antibody was validated by the lack of signal in *indra^RNAi^* testis (fig. S2A). Alexa Fluor–conjugated secondary antibodies (Life Technologies) were used at a dilution of 1:200. Images were taken on a Leica TCS SP8 confocal microscope with a 63× oil immersion objective [numerical aperture (NA) = 1.4] and processed using Adobe Photoshop software.

For DNA FISH combined with immunofluorescent staining, whole-mount *Drosophila* testes were prepared as described above, and the immunofluorescence staining protocol was carried out first. Upon completion of the wash after incubation with the secondary antibody, samples were fixed with 4% formaldehyde for 10 min and washed in PBST for 30 min. Fixed samples were incubated with ribonuclease A (RNase A) solution (2 mg/ml) at 37°C for 10 min and then washed with PBST. Samples were washed in 2× saline sodium citrate (SSC) with increasing formamide concentrations (20 and 50%) for 10 min each. Hybridization buffer (50% formamide, 10% dextran sulfate, 2× SSC, 1 mM EDTA, and 1 mM probe) was added to washed samples. Samples were denatured at 91°C for 2 min and then incubated overnight at 37°C. Following the hybridization, testes were washed once in 50% formamide/2× SSC, once in 20% formamide/2× SSC, and three times in 2× SSC. All reagents contained 1 mM EDTA except for the washes before the RNase A treatment. Fluorescence quantification was done on merged z stacks using ImageJ “Sum of pixel intensity (RawIntDen)” to compare signal intensity between sister chromatids. To avoid the effect of signal intensity changes along the *Z* plane, we scored anaphase GSCs only when two IGS FISH signals were found within the same *Z* plane for Fig. 4 (C to E).

### Chromosome orientation fluorescence in situ hybridization

CO-FISH in whole-mount *Drosophila* testes was performed as previously described (*7*). Briefly, young adult flies (days 1 to 3) were fed with 5-bromodeoxyuridine (BrdU)–containing food [950 μl of 100% apple juice, 7 mg of agar, and 50 μl of BrdU solution (100 mg/ml) in a 1:1 mixture of acetone and dimethyl sulfoxide] for 12 hours. After the feeding period, flies were transferred to regular fly food for 13.5 hours. Because the average GSC cell cycle length is ~12 hours, most GSCs undergo a single S phase in the presence of BrdU, followed by mitosis during this feeding procedure. GSCs that have undergone more or less than one S phase or mitosis were excluded from our analysis by limiting the scoring to GSC-GB pairs that have complementary CO-FISH signals in the GSC and GB (red signal in one cell and blue signal in the other). Note that GSC and GB stay connected by the fusome until mid–S phase, which allowed identification of the GSC-GB sister pairs. Testes were dissected, fixed, and immunostained as described above. Then, testes were fixed for 10 min with 4% formaldehyde in PBS, followed by three washes with PBST. Following the washes, the testes were rinsed once with PBST and treated with RNase A (2 mg/ml in PBS; Roche) for 10 min at 37°C, washed with PBST for 5 min, and stained with 100 μl of Hoechst 33258 (2 μg/ml; Invitrogen) in 2× SSC for 15 min at room temperature. The testes were then rinsed three times with 2× SSC, transferred to a tray, and irradiated with ultraviolet light in a CL-1000 Ultraviolet Crosslinker (Ultra-Violet Products Ltd.; wavelength, 365 nm; calculated dose, 5400 J/m^2^). Nicked BrdU-positive strands were digested with exonuclease III (New England Biolabs) at 3 U/μl in 1× NEBuffer 1 or 1× NEB CutSmart buffer for 10 min at 37°C. The testes were washed once with PBST for 5 min and then fixed with 4% formaldehyde in PBS for 2 min. Subsequently, the fixed testes were washed three times with PBST. Testes were incubated sequentially for a minimum of 10 min each in 20% formamide/2× SSC and 50% formamide/2× SSC. The testes were incubated with hybridization buffer (50% formamide, 2× SSC, and 10% dextran sulfate) containing 1 μM of each probe for 16 hours at 37°C. Following hybridization, testes were washed once in 50% formamide/2× SSC, once in 20% formamide/2× SSC, and three times in 2× SSC. Images were taken on either a Leica TCS SP5 or STELLARIS 8 confocal microscope with a 63× oil immersion objective (NA = 1.4) and processed using Adobe Photoshop software. For CO-FISH in GSCs from *tub-gal80^ts^*; *nos>indra^TRiP.HMJ30228^*, the BrdU pulse was conducted 3 days after temperature shift (fig. S2C). BrdU was fed at 29°C for 9 hours, followed by an 11-hour chase at 29°C, in which the flies were fed regular fly food. The probes are described in table S6. All reagents contained 1 mM EDTA except for the washes immediately preceding an enzymatic reaction (RNase A and exonuclease III).

### IGS DNA pull down and MS

A total of 200 pairs of *upd*-expressing testes (*nos-gal4>UAS-upd*) were dissected in Schneider’s *Drosophila* Medium (Gibco) and washed three times with ice-cold PBS. *upd* expression causes overproliferation of GSC-like cells. The testes were homogenized in lysis buffer [20 mM tris-HCl (pH 8.0), 1 mM EDTA, 10% glycerol, 0.2% NP-40, 1 mM dithiothreitol (DTT), 1× solution of PhosSTOP cocktail (Roche), and 1× solution of cOmplete EDTA-free protease inhibitor cocktail (Roche)], and the homogenate was incubated on ice for 20 min. Following this incubation, the lysate was centrifuged at 3000 rpm for 10 min at 4°C, and the supernatant was saved as a whole-cell extract. The pellet, which contains the nuclear fraction, was resuspended in lysis buffer containing 100 mM NaCl and incubated on ice for 1 hour. During incubation, the sample was vortexed at highest setting for 15 s every 10 min. The nuclear fraction was isolated by centrifugation at 14,000 rpm for 30 min at 4°C and mixed with the whole-cell extract prepared above. Protein concentration was measured by absorbance at 562 nm using the Pierce BCA Protein Assay Kit (Thermo Fisher Scientific) to adjust the concentration between samples.

The 240-bp IGS sequence (repeated four times, 4× IGS), or the *Kr* gene promoter sequence (control), was cloned into pBluescript SK^−^. Biotin end labeling at the 5′ of one strand of the 4× IGS or *Kr* gene promoter was performed by PCR using a T7 primer with Biotin-TEG and a T3 primer. Biotinylated 4× IGS and *Kr* gene promoter DNA were then purified by QIAGEN’s PCR purification kit. Two micrograms of each biotinylated DNA was immobilized to 100 μl of streptavidin-bound M-280 Dynabeads (Invitrogen). The beads were washed three times with 1× binding and washing buffer [5 mM tris-HCl (pH 8.0), 0.5 mM EDTA, and 500 mM NaCl] and then blocked with 0.5% BSA in TGEDN buffer [120 mM tris-HCl (pH 8.0), 1 mM EDTA, 100 mM NaCl, 1 mM DTT, 0.1% Triton X-100, and 10% glycerol]. Twenty percent volume of each of the biotinylated DNA-conjugated Dynabeads was incubated with 20 μg of herring sperm DNA (Sigma-Aldrich) and the cell extract prepared above [containing 500 μg of protein at concentration (3.8 to 4.8 μg/μl), matched between control and IGS beads]. After incubating for 2 hours at 4°C, the beads were washed five times with TGEDN buffer. The proteins bound to either the 4× IGS or *Kr* gene promoter DNA were eluted in LDS sample loading buffer (1.5×) at 100°C for 15 min. Fifty percent volume of each DNA bound proteins was separated on a 10% bis-tris Novex mini-gel (Invitrogen) using the MES buffer system. The gel was stained with Coomassie and excised into 10 equally sized segments. These segments were analyzed by liquid chromatography–tandem MS (LC-MS/MS) (MS Bioworks, Ann Arbor, MI). The gel digests were analyzed by nano LC-MS/MS with a Waters nanoACQUITY HPLC system interfaced to a Thermo Fisher Scientific Q Exactive. Peptides were loaded on a trapping column and eluted over a 75-μm analytical column at 350 nl/min; both columns were packed with Luna C18 resin (Phenomenex). The mass spectrometer was operated in data-dependent mode, with MS and MS/MS performed in the Orbitrap at 70,000 and 17,500 full width at half maximum resolution, respectively. The 15 most abundant ions were selected for MS/MS.

### Chromatin immunoprecipitation quantitative polymerase chain reaction

A total of 200 pairs of *upd*-expressing testes (*nos-gal4>UAS-upd*) were dissected in ice-cold PBS containing protease inhibitor [1× solution of cOmplete protease inhibitor cocktail (Roche) and 1 mM phenylmethylsulfonyl fluoride]. The testes were cross-linked by incubating with 1% formaldehyde for 15 min at 37°C and rinsed twice in ice-cold PBS containing protease inhibitor to stop the cross-link reaction. The testes were homogenized in 200 μl of ice-cold ChIP sonication buffer [1% Triton X-100, 0.1% sodium deoxycholate, 50 mM tris-HCl (pH 8.0), 150 mM NaCl, and 5 mM EDTA], and the homogenate was incubated on ice for 15 min. Following the incubation, the homogenate was aliquoted into 0.5-ml PCR tubes, placed in a Bioruptor Plus sonication system (Diagenode), and sonicated in 4°C water bath for 10 cycles of 30 s “on” and 30 s “off” at “high” setting. The sonicated lysate was centrifuged at 14,000 rpm for 10 min at 4°C to pellet cell debris. The volume of supernatant was brought up to 1 ml with ChIP sonication buffer, and 40 μl of Dynabeads Protein A (Invitrogen) was added to the supernatant. After a 1-hour preabsorption with Dynabeads Protein A at 4°C, 30 μl of supernatant (3%) was kept as “input.” The rest was split into two and incubated overnight with 10 μl of anti-Indra antibody (1:10 dilution from the original serum; generated as described above) or 10 μl of preimmune guinea pig serum (1:10 dilution from the original serum), respectively. After incubating for 16 hours, 40 μl of Dynabeads Protein A was added to each reaction and incubated for an additional 4 hours at 4°C with rotation. The beads were then washed for 5 min at 4°C with 1 ml of the following buffers: two washes with ChIP sonication buffer, followed by three washes with high-salt wash buffer [1% Triton X-100, 0.1% sodium deoxycholate, 50 mM tris-HCl (pH 8.0), 500 mM NaCl, and 5 mM EDTA], two washes with LiCl Immune Complex Wash buffer [250 mM LiCl, 0.5% NP-40, 0.5% deoxycholate, 1 mM EDTA, and 10 mM tris-HCl (pH 8.0)], and one wash with TE buffer [10 mM tris-HCl (pH 8.0) and 1 mM EDTA]. For elution, each ChIP sample was incubated with 250 μl of elution buffer (1% SDS and 100 mM NaHCO_3_) for 30 min at 65°C, vortexing gently every 10 min. After repeating the elution process once more, the supernatants were combined. A total of 500 μl of elution buffer was added to the input sample. Twenty microliters of 5 M NaCl and 10 μl of RNase A [2 mg/ml in 10 mM tris-HCl (pH 7.5) and 15 mM NaCl; Roche] were added to each sample and incubated for overnight at 65°C. After incubating for 16 hours, 2 μl of proteinase K (New England Biolabs), 10 μl of 500 mM EDTA, and 20 μl of 1 M tris-HCl (pH 8.0) were added to each sample and incubated at 45°C for 2 hours. The precipitated DNA was purified using QIAGEN’s PCR purification kit. Real-time PCR was conducted to quantify precipitated DNA using the standard curve method. Power SYBR Green PCR Master Mix (Applied Biosystems) was used as the PCR reaction buffer. The QuantStudio 6 Flex System (Applied Biosystems) was used for real-time PCR reaction and analyzing the data. Primers used for real-time PCR are listed in table S7.

### Fertility assay

Newly eclosed single males [control (*nos-gal4*) or *nos-gal4>UAS-indra^TRiP.HMJ30228^*] were individually crossed to three *yw* virgin females. After 5 days, each male was transferred to a new vial with three new virgin females. The number of adult flies that eclosed in each vial was scored. *nos-gal4>UAS-indra^TRiP.HMJ30228^* females are completely sterile. Therefore, to examine the fertility of *nos>indra^TRiP.HMJ30228^* across generations, newly eclosed *nos-gal4>UAS-indra^TRiP.HMJ30228^* males for each generation were crossed to *nos-gal4* females to deplete *indra* in the germ line in the subsequent generation. Then, newly eclosed single males [control (*nos-gal4*) or *nos-gal4>UAS-indra^TRiP.HMJ30228^*] at each generation were individually crossed to three *yw* virgin females, and the number of adult flies that eclosed in each vial was scored.

### Droplet digital PCR

Twenty pairs of testes/sample were dissected from 0- to 3-day-old control (*nos-gal4*) or *nos-gal4>UAS-indra^TRiP.HMJ30228^* males. Genomic DNA isolation was performed as previously described (*36*). Briefly, the testes were homogenized in 200 μl of buffer A [100 mM tris-HCl (pH 8.0), 100 mM EDTA (pH 8.0), 100 mM NaCl, and 0.5% SDS], and then an additional 200 μl of buffer A were added to the homogenate. The homogenate was incubated at 65°C for 30 min. Then 800 μl of LiCl/KAc (2.5:1 mixture of 6 M LiCl and 5 M KAc) was added to the homogenate, and the sample was left on ice for 15 min. Subsequently, the sample was centrifuged at 14,000 rpm for 15 min, and 1 ml of supernatant was transferred to a new tube. The supernatant was mixed with 600 μl of isopropanol and centrifuged at 14,000 rpm for 15 min. The pellet (containing genomic DNA) was washed once in 1 ml of 70% ethanol, air-dried for 30 min, and dissolved in 35 μl of TE buffer. The quality and concentration of genomic DNA were measured on a NanoDrop One (Thermo Fisher Scientific).

Thirty nanograms of genomic DNA was used per 20-μl ddPCR reaction for control gene reactions (RpL and Upf1), and 0.3 ng of genomic DNA was used per 20-μl ddPCR reaction for 28*S* rRNA gene reactions. The primers and probes are listed in table S7. ddPCR reactions were carried out according to the manufacturer’s protocol (Bio-Rad). Briefly, master mixes containing ddPCR Supermix for Probes (no deoxyuridine triphosphate nick end labeling) (Bio-Rad), genomic DNA, primer/probe mixes, and HindIII-HF restriction enzyme (New England Biolabs) for 28*S* rRNA gene reactions (no restriction enzyme is needed for the control gene reactions) were prepared in 0.2-ml PCR tubes and incubated at room temperature for 15 min to allow for restriction enzyme digestion. ddPCR droplets were generated from samples using a QX200 Droplet Generator (Bio-Rad), and droplets then underwent complete PCR cycling on a C100 deep-well thermocycler (Bio-Rad). Droplet fluorescence was read using a QX200 droplet reader (Bio-Rad). Sample copy number was determined using QuantaSoft software (Bio-Rad). rDNA copy number per genome was determined by 28*S* copy number multiplied by 100 (due to the 100× dilution of genomic DNA in the 28*S* reaction compared to control reaction), divided by control gene copy number, and multiplied by the expected number of control gene copies per genome (two for RpL samples and one for Upf1 samples). The 28*S* rRNA gene copy number values normalized by each control were then averaged to determine 28*S* copy number for each sample.

### Magnification assay

The experimental design to assay rDNA magnification is shown in fig. S5. The *bb^z9^* allele carries an insufficient rDNA copy number on the X chromosome (*37*), which exhibits a “*bobbed*” cuticle phenotype when combined with the *bb^158^* allele (no rDNA on X chromosome) in females. To induce magnification, the *bb^z9^* allele was combined with a Y chromosome lacking rDNA (*bb^z9^/Ybb^−^*) (magnifying condition). These *bb^z9^/Ybb^−^* males were crossed to *bb^158^/FM6* females, and the resulting *bb^z9^/bb^158^* females were examined for the *bobbed* cuticle phenotype. If magnification occurred, then a magnified allele (*bb^z9-mag^*) combined with *bb^158^* would produce a wild-type cuticle, whereas a nonmagnified allele combined with *bb^158^* would show a *bobbed* cuticle phenotype. The frequency of wild-type cuticle among total female progeny without FM6 (i.e., *bb^z9^* and *bb^z9-mag^* / *bb^158^*) was scored as “magnification frequency.”

### Statistical analysis

For comparison of sister chromatid segregation patterns in Figs. 1 (C and D), 2F, and 4G and fig. S1, significance was determined by two-sided Fisher’s exact tests. For comparison of frequencies of *bobbed* animals in Fig. 3B and wild-type cuticle animals in Fig. 3E, significance was determined by two-tailed chi-squared tests using a 2 × 2 contingency table (normal and *bobbed*). Other than these, significance was determined by two-tailed Mann-Whitney tests.

## Supplementary Materials

### This PDF file includes:

Figs. S1 to S6 Tables S1 to S7 References

## Notes

View/request a protocol for this paper from *Bio-protocol*.
