# Supplementary material for: Nonrandom sister chromatid segregation mediates rDNA copy number maintenance in Drosophila
Source: Sci Adv. 2022 Jul 27;8(30):eabo4443. doi: 10.1126/sciadv.abo4443 (PMC9328678; doi:10.1126/sciadv.abo4443)
Supplement: Supplementary file 1 — Figs. S1 to S6 Tables S1 to S7 References [file sciadv.abo4443_sm.pdf]

Science Advances
AAAS

Supplementary Materials for

Nonrandom sister chromatid segregation mediates rDNA copy number maintenance in Drosophila

George J. Watase *et al.*

Corresponding author: Yukiko M. Yamashita, yukikomy@wi.mit.edu

Sci. Adv. **8**, eabo4443 (2022)
DOI: 10.1126/sciadv.abo4443

This PDF file includes:

Figs. S1 to S6
Tables S1 to S7
References

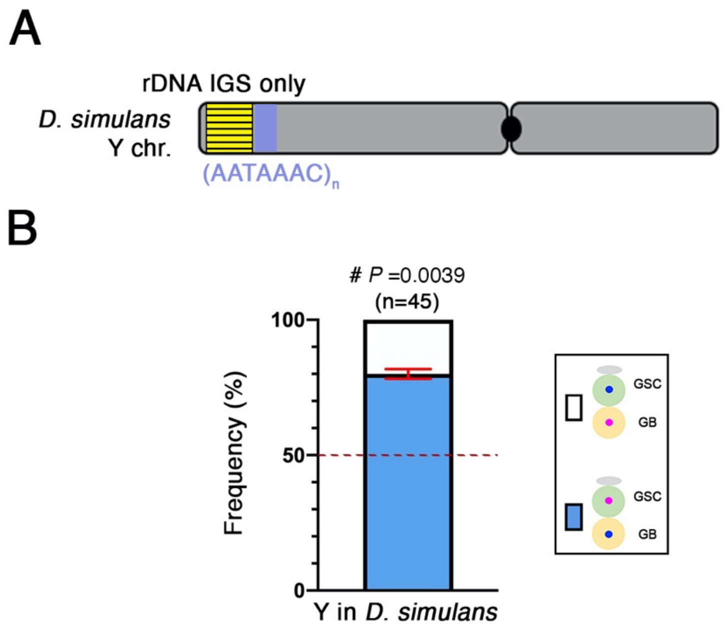

Fig. S1. The *D. simulans* Y chromosome segregates sister chromatids non-randomly

(A) Schematic of the *D. simulans* Y chromosome

(B) Summary of the sister chromatid segregation pattern assessed by CO-FISH in the *D. simulans* control strain (w^{501}) (see table S1 for detailed data). Data shown as mean ± s.d. from three independent experiments. n, number of GSC-GB pairs scored. #, *P*-value of Fisher's exact test by comparing to hypothetical random sister chromatid segregation is shown.

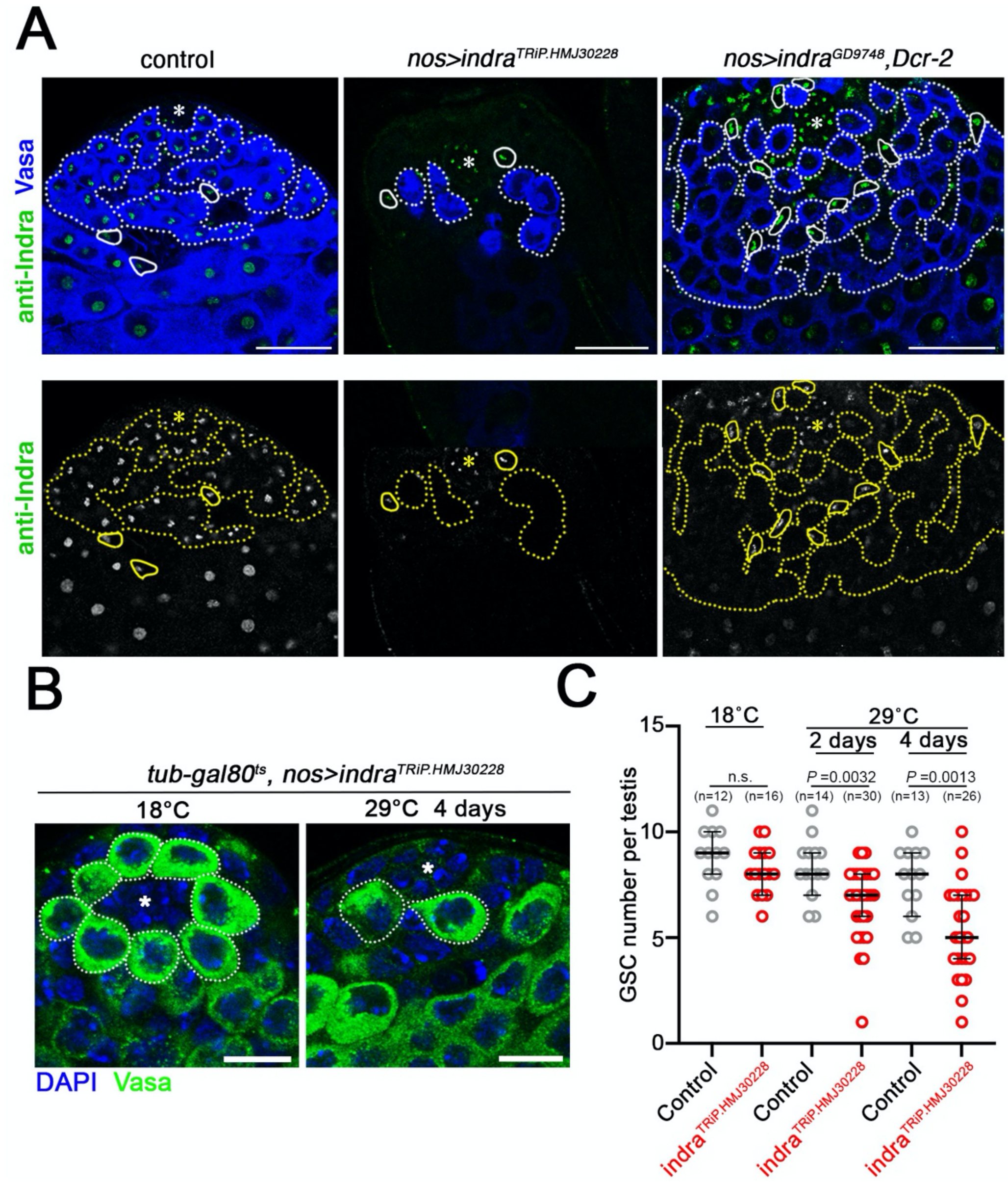

Fig. S2. Validation of *indra*^{RNAi} efficiency and antibody specificity

(A) Examples of testis apical tips after *indra* knockdown by indicated *indra*^{RNAi} lines. Anti-Indra antibody staining was lost from germ cells upon *indra* knockdown by *nos-gal4>indra*$^{TRiP.HMJ30228}$ or *nos-gal4>indra*^{GD9748}*, Dcr-2*. (UAS-Dcr-2 was added to enhance the efficiency of *indra*^{GD9748}). This experiment also demonstrates the specificity of the anti-Indra antibody. The hub is indicated by an asterisk. Germ cells are indicated by dotted lines and somatic cells are indicated by solid lines. Bar: 25 μm.

(B) Examples of testis apical tips before and after induction of *nos-gal4>indra*$^{TRiP.HMJ30228}$. GSCs are indicated by dotted lines and the hub is indicated by an asterisk. Bar: 10 μm.

(C) GSC number after induction of *indra*$^{TRiP.HMJ30228}$. n, number of testes scored. *P*-values: two-tailed Mann-Whitney test.

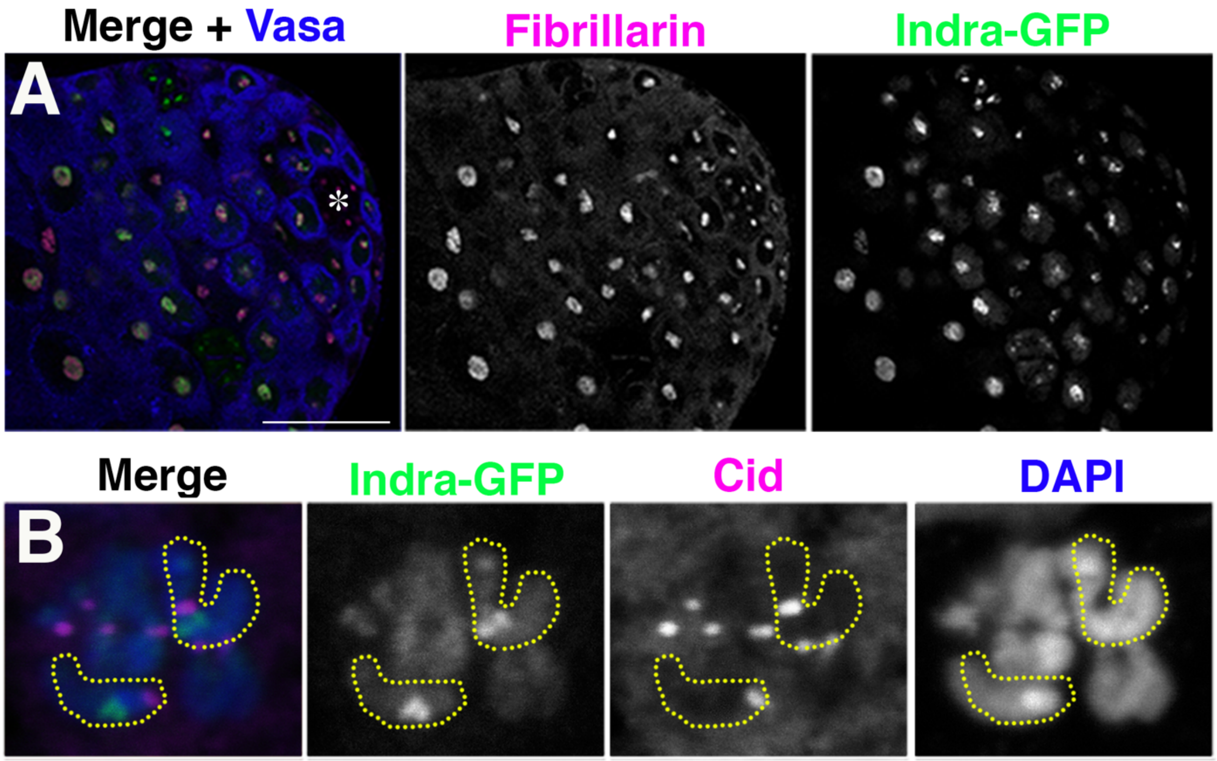

Fig. S3. Localization of Indra-GFP to the nucleolus and rDNA loci

a) Localization of Indra-GFP at the apical tip of the testis. Indra localizes to nucleolus visualized by Fibrillarin. The hub is indicated by an asterisk. Bar: 25 µm.

b) Localization of Indra-GFP on a metaphase chromosome spread. The X and Y chromosomes are indicated by dotted lines. Cid: centromere. Bar: 5 µm.

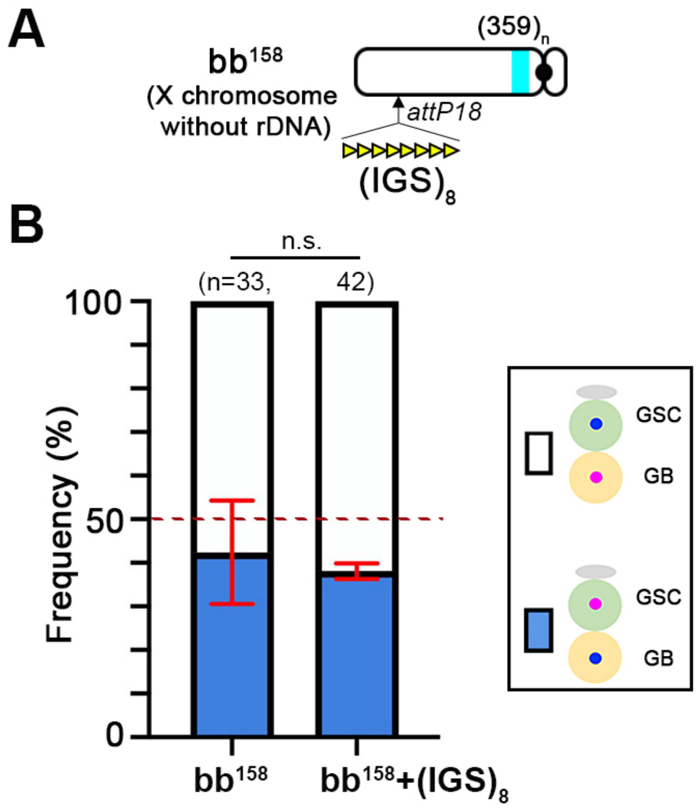

Fig. S4. Insertion of 8 copies of IGS to X chromosome without rDNA is not sufficient to restore NRSS

(A) Schematic of the bb^{158} chromosome with insertion of 8 copies of IGS.

(B) Summary of sister chromatid segregation pattern assessed by CO-FISH in the bb^{158} and bb^{158} with 8 copies of IGS insertion. (see table S1 for detailed data). Data shown as mean ± s.d. from three independent experiments. n, number of GSC-GB pairs scored. *P*-value: Fisher's exact test.

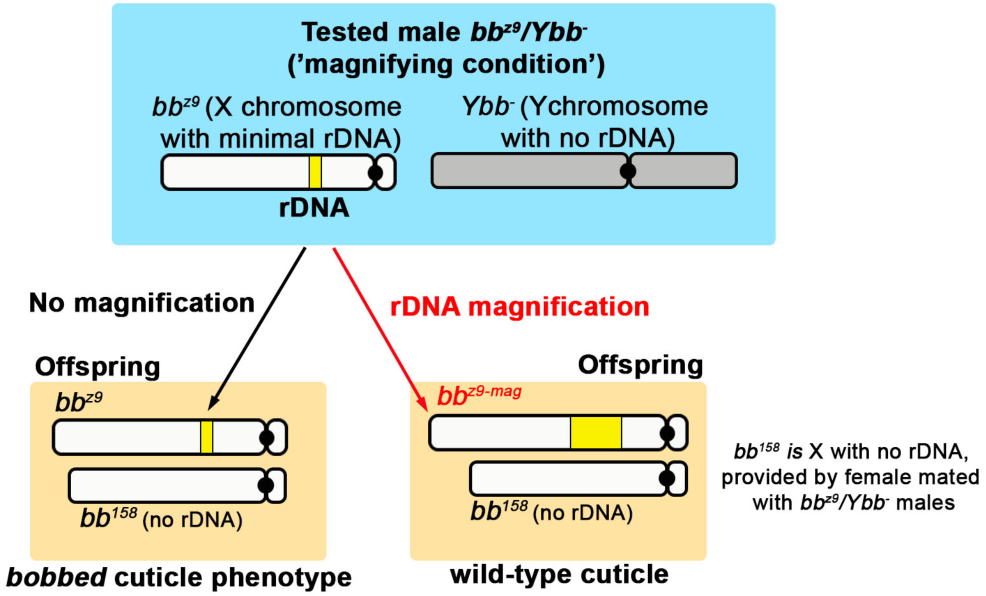

Fig. S5. Diagram of phenotypic assessment to detect rDNA magnification of the bb^{z9} allele

The *bb^{z9}* allele carries an insufficient rDNA copy number on the X chromosome, which causes flies to exhibit a '*bobbed*' cuticle phenotype when combined with the *bb^{158}* allele (no rDNA on X chromosome) in females (Fig. 3B). To induce magnification, the *bb^{z9}* allele was combined with a Y chromosome without rDNA (*bb^{z9}/Ybb^{-})*('magnifying condition'). To assess whether the *bb^{z9}* allele magnified, these *bb^{z9}/Ybb^{-}* males were crossed to *bb^{158}/FM6* female, and cuticle phenotype of the resulting *bb^{z9}/bb^{158}* females was examined. If magnification occurred, the magnified allele (*bb$^{z9\text{-}mag}$*) combined with *bb^{158}* would have a wild type cuticle, whereas the non-magnified allele combined with *bb^{158}* would have the *bobbed* phenotype. The frequency of wild type cuticle among total female progeny without FM6 (i.e. *bb^{z9}* and *bb$^{z9\text{-}mag}$/bb^{158}*) was scored as 'magnification frequency'.

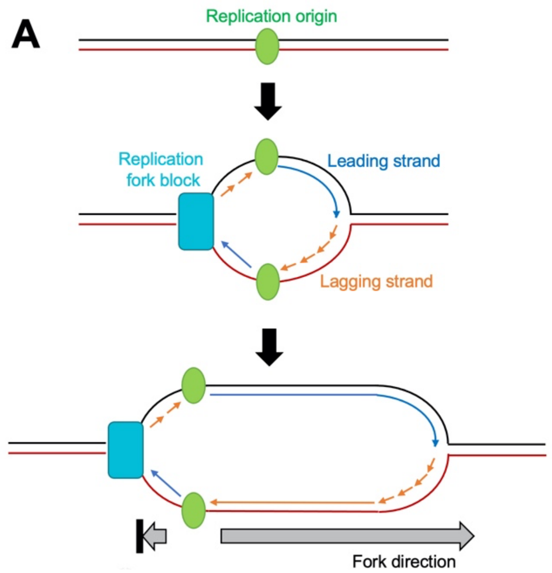

Fig. S6. Diagram of DNA replication at rDNA loci

(A) Replication fork block on one side of the replication origin leads to mostly unidirectional DNA replication at the rDNA loci. This causes one sister chromatid to be synthesized primarily as leading strand and the other as lagging strand.

(B) In yeast, double strand DNA breaks primarily occur on the leading strand when fork progression is prevented at the replication fork block (top). An appropriate donor for DNA repair may be found in the region of the sister chromatid replicated as leading strand. If such recombination happens, the sister chromatid mostly replicated as lagging strand (bottom strand) will gain copy number.

Table S1. CO-FISH results in *D. melanogaster* rDNA deficient stocks and *D. simulans*

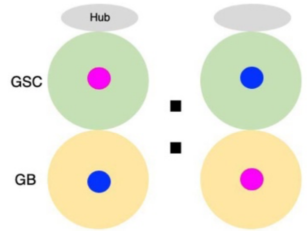

		Outcome	
		**Y chromosome**	**X chromosome**
D. melanogaster	wild type (*yw)*	76.9%:23.1% (±2.2%) (n=299)	77.4%:22.6% (±1.0%) (n=124)
	Df(1)bb^{158}/Y	76.2%:23.8% (±3.5%) (n=63)	42.4%:57.6% (±11.9%) (n=33)
	Df(1)bb^{158} with $(IGS)_8$/Y	N.D.	38.1%:61.9% (±1.8%) (n=42)
	X/Df(YS)bb$^-$	45.7%:54.3% (±8.7%) (n=46)	75.5%:24.5% (±5.7%) (n=53)
D. simulans	wild type (*w*^{501})	80.0%:20.0% (±1.8%) (n=45)	N.D.

Probes used:
D. mel Y chromosome: Cy3-$(AATAC)_6$, Cy5-$(GTATT)_6$
D. mel X chromosome: Cy3-359 forward, Cy5-359 reverse
D. sim Y chromosome: Cy5-$(GTTTATT)_6$, Cy3-$(AATAAAC)_6$

Table S2. CO-FISH results of Y-2 translocation chromosomes

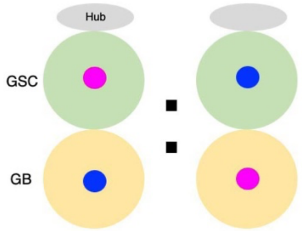

		Outcome
		**T(Y;2) chromosome**
T(Y;2)A77/+; XO	2^Y (with rDNA)	79.3%:20.7% (±0.5%) (n=58)
	Y^2	N.D.
T(Y;2)P8/+; XO	2^Y	46.6%:53.4% (±5.8%) (n=73)
	Y^2 (with rDNA)	78.1%:21.9% (±9.3%) (n=32)

Probes used:
T(Y;2) chromosome 2^Y: Cy5-$(GTTTATT)_6$, Cy3-$(AATAAAC)_6$
T(Y;2) chromosome Y^2: Cy3-$(AATAC)_6$, Cy5-$(GTATT)_6$

Table S3. List of proteins that were enriched in IGS-beads pull-down

Gene Name	Experiment 1	Experiment 2	Localized to nucleolus?	Localized to rDNA in mitosis?
Rrp1	19/0	7/0	N.D.	N.D.
CG2199/Indra	8/0	2/0	**YES**	**YES**
Iswi	8/0	0/0	**YES** (*38*)	N.D.
D1	60/3	37/0	**NO**	**NO**
apt	9/0	13/0	N.D.	N.D.
IleRS	26/0	11/0	**NO** (*39*)	N.D.
Dp1	21/0	9/0	N.D.	N.D.
dre4	21/0	9/0	**(YES)***	**(NO)***
Dsp1	14/0	8/0	**YES**	**NO**
clu	15/0	7/0	**NO** (*40*)	N.D.
Hrb27C	14/0	6/0	**NO** (*41*)	N.D.
TppII	20/0	6/0	**NO** (*42*)	N.D.
Prosα3	6/0	5/0	N.D.	N.D.
l(2)37Cc	14/2	5/0	**NO** (*43*)	N.D.
Cyt-c-p	9/0	5/0	**NO** (*44*)	N.D.
RpL8	9/2	5/0	**YES** (*45*)	N.D.
CG3995	5/0	5/0	**NO**	**NO**
TFAM	25/4	24/4	**NO** (*46*)	N.D.

* As no reagents to visualize the localization of dre4, which is a component of the FACT complex, were available, the localization of SSRP1 (another component of the FACT complex) was used when deciding whether or not to follow up dre4 in this study.

Data are shown as peptide counts in IGS beads/control beads.

Table S4. CO-FISH results upon knockdown of *indra*

	Outcome<br>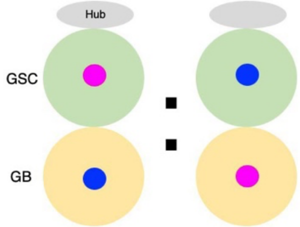	
	**Y chromosome**	**X chromosome**
indra$^{TRiP.HMJ30228}$ control*	75.0%:25.0% (±2.6%) (n=40)	80.0%:20.0% (±1.7%) (n=30)
tub-gal80^{ts}, *nos-gal4ΔVP16>* *UAS-indra*^{HMJ30228}	40.0%:60.0% (±0.0%) (n=30)	43.6%:56.4% (±9.6%) (n=39)
indra^{GD9748} control (*nos-gal4>UAS-Dcr-2*)	76.9%:23.1% (±7.4%) (n=26)	76.9%:23.1% (±7.9%) (n=39)
nos-gal4> *UAS-indra*^{GD9748}, *UAS-Dcr-2*	42.9%:57.1% (±2.1%) (n=28)	43.2%:56.8% (±7.5%) (n=44)

Probes used:
Y chromosome: Cy3-(AATAC)$_6$, Cy5-(GTATT)$_6$
X chromosome: Cy3-359 forward, Cy5-359 reverse
*: Cross siblings of *tub-gal80*^{ts}*, nos-gal4ΔVP16>UAS-indra*$^{TRiP.HMJ30228}$ that do not express *indra*^{HMJ30228} (either *nos-gal4ΔVP16* only or *UAS-indra*$^{TRiP.HMJ30228}$ only) were used as control

Table S5. CO-FISH results of X chromosome in magnifying condition

		Outcome<br>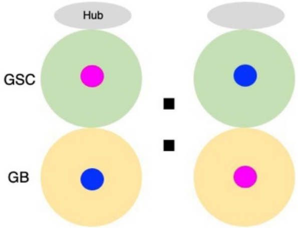
		**X chromosome**
Non-magnifying (*yw*)	TAGA	87.5%:12.5% (±11.3%) (n=32)
	359	81.1%:18.9% (±0.9%) (n=37)
Magnifying (bb^{z9}/Ybb^{-})	TAGA	53.3%:46.7% (±4.7%) (n=45)
	359	74.2%:25.8% (±7.7%) (n=31)

Probes used:
(TAGA)$_n$: Cy3-(TAGA)$_8$, Cy5-(TCTA)$_8$
(359)$_n$: Cy3-359 forward, Cy5-359 reverse

Table S6. Probe sequences for CO-FISH and DNA FISH

Probe target	5'-sequence-3'	Source or reference	Related figure
$(AATAC)_n$ (forward)	Cy3-$(AATAC)_6$	(*7*)	Fig. 1, B-D, Fig. 2F
$(AATAC)_n$ (reverse)	Cy5-$(GTATT)_6$		Fig. 1, B-D, Fig. 2F
359-bp (forward)	Cy3-CCACATTTTGCAAATTTTGATGACCCCCCTCCTTACAAAAAATGCG		Fig. 1C, Fig. 2F, Fig. 4G
359-bp (reverse)	Cy5-AGGATTTAGGGAAATTAATTTTTGGATCAATTTTCGCATTTTTTGTAAG		Fig. 1C, Fig. 2F, Fig. 4G
$(AATAAAC)_n$ (forward)	Cy3-$(AATAAAC)_6$	This study	Fig. 1D, fig. S1B
$(AATAAAC)_n$ (reverse)	Cy5-$(GTTTATT)_6$	(*47*)	Fig. 1D, fig. S1B
$(TAGA)_n$ (forward)	Cy3-$(TAGA)_8$	(*47*)	Fig. 4G
$(TAGA)_n$ (reverse)	Cy5-$(TCTA)_8$	This study	Fig. 4G
240-bp IGS	Cy5-TCCATTCACTAAAATGGCTTTTCTCTATAATACTTAGAGAATATGGGAATATTTCAACATTTTTCACT	(*17*)	Fig. 4, C-E

Table S7: Primer and probe sequences for Real-Time PCR and Droplet-Digital PC

**Primer name**	**5'-sequence-3'**	**Source or reference**	**Related figure**
rt-5S rDNA (forward)	AAGTTGTGGACGAGGCCAAC	(*48*)	Fig. 2E
rt-5S rDNA (reverse)	CGGTTCTCGTCCGATCACCGA		
rt-IGS #1 (forward)	GCTGTTCTACGACAGAGGGTTC		
rt-IGS #1 (reverse)	CAATATGAGAGGTCGGCAACCAC		
rt-IGS #2 (forward)	GGTAGGCAGTGGTTGCCG		
rt-IGS #2 (reverse)	GGAGCCAAGTCCCGTGTTC		
rt-ETS (forward)	ATTACCTGCCTGTAAAGTTGG		
rt-ETS (reverse)	CCGAGCGCACATGATAATTCTTCC		
rt-18S rDNA (forward)	TTCTGGTTGATCCTGCCAGTAG		
rt-18S rDNA (reverse)	CGTGTGTACTTAGACATGCATGGC		
rt-28S rDNA (forward)	CCTCAACTCATATGGGACTACC	This study	
rt-28S rDNA (reverse)	CACTGCATCTCACATTTGCC		
dd-RpL32 (forward)	GCTTCAAGGGACAGTATCTG	(*37*)	Fig. 3C
dd-RpL32 (reverse)	AACGCGGTTCTGCATGAG		
dd-RpL32 (probe)	**HEX**-ATGCCCAACATCGGTTAC-**Iowa Black FQ**		
dd-28S (forward)	GAGCTGCCATTGGTACAG		
dd-28S (reverse)	GCTTTCGCCTTGAACTTAG		
dd-28S (probe)	**HEX**-TGGTGGATAGTAGCAAATAATCG-**Iowa Black FQ**		
dd-Upf1 (forward)	CACACTTTATGTCCACCATTATTG		
dd-Upf1 (reverse)	GAGTTTCCGTAGGGACCAC		
dd-Upf1 (probe)	**HEX**-CCGTAACCGCCACTGCGGT-**Iowa Black FQ**		

REFERENCES AND NOTES

1. P. M. Lansdorp, Immortal strands? Give me a break. *Cell* **129**, 1244–1247 (2007).

2. T. A. Rando, The immortal strand hypothesis: Segregation and reconstruction. *Cell* **129**, 1239–1243 (2007).

3. S. Tajbakhsh, C. Gonzalez, Biased segregation of DNA and centrosomes: Moving together or drifting apart? *Nat. Rev. Mol. Cell Biol.* **10**, 804–810 (2009).

4. C. S. Potten, W. J. Hume, P. Reid, J. Cairns, The segregation of DNA in epithelial stem cells. *Cell* **15**, 899–906 (1978).

5. P. M. Lansdorp, E. Falconer, J. Tao, J. Brind'Amour, U. Naumann, Epigenetic differences between sister chromatids? *Ann. N. Y. Acad. Sci.* **1266**, 1–6 (2012).

6. E. H. Zion, C. Chandrasekhara, X. Chen, Asymmetric inheritance of epigenetic states in asymmetrically dividing stem cells. *Curr. Opin. Cell Biol.* **67**, 27–36 (2020).

7. S. Yadlapalli, Y. M. Yamashita, Chromosome-specific nonrandom sister chromatid segregation during stem-cell division. *Nature* **498**, 251–254 (2013).

8. S. Tajbakhsh, Stem cell identity and template DNA strand segregation. *Curr. Opin. Cell Biol.* **20**, 716–722 (2008).

9. R. S. Hawley, C. H. Marcus, Recombinational controls of rDNA redundancy in *Drosophila*. *Annu. Rev. Genet.* **23**, 87–120 (1989).

10. A. R. Lohe, P. A. Roberts, An unusual Y chromosome of *Drosophila* simulans carrying amplified rDNA spacer without rRNA genes. *Genetics* **125**, 399–406 (1990).

11. F. M. Ritossa, K. C. Atwood, S. Spiegelman, A molecular explanation of the bobbed mutants of *Drosophila* as partial deficiencies of "ribosomal" DNA. *Genetics* **54**, 819–834 (1966).

12. K. D. Tartof, Unequal mitotic sister chromatin exchange as the mechanism of ribosomal RNA gene magnification. *Proc. Natl. Acad. Sci. U.S.A.* **71**, 1272–1276 (1974).

13. K. D. Tartof, Unequal mitotic sister chromatid exchange and disproportionate replication as mechanisms regulating ribosomal RNA gene redundancy. *Cold Spring Harb. Symp. Quant. Biol.* **38**, 491–500 (1974).

14. T. Kobayashi, Ribosomal RNA gene repeats, their stability and cellular senescence. *Proc. Jpn. Acad. Ser. B Phys. Biol. Sci.* **90**, 119–129 (2014).

15. A. Bianciardi, M. Boschi, E. E. Swanson, M. Belloni, L. G. Robbins, Ribosomal DNA organization before and after magnification in *Drosophila melanogaster*. *Genetics* **191**, 703–723 (2012).

16. S. A. Endow, D. J. Komma, K. C. Atwood, Ring chromosomes and rDNA magnification in *Drosophila*. *Genetics* **108**, 969–983 (1984).

17. K. L. Lu, J. O. Nelson, G. J. Watase, N. Warsinger-Pepe, Y. M. Yamashita, Transgenerational dynamics of rDNA copy number in *Drosophila* male germline stem cells. *eLife* **7**, e32421 (2018).

18. F. M. Ritossa, Unstable redundancy of genes for ribosomal RNA. *Proc. Natl. Acad. Sci. U.S.A.* **60**, 509–516 (1968).

19. D. V. de Cicco, D. M. Glover, Amplification of rDNA and type I sequences in *Drosophila* males deficient in rDNA. *Cell* **32**, 1217–1225 (1983).

20. T. Akera, E. Trimm, M. A. Lampson, Molecular strategies of meiotic cheating by selfish centromeres. *Cell* **178**, 1132–1144.e10 (2019).

21. R. Ranjan, J. Snedeker, X. Chen, Asymmetric centromeres differentially coordinate with mitotic machinery to ensure biased sister chromatid segregation in germline stem cells. *Cell Stem Cell* **25**, 666–681.e5 (2019).

22. Y. Akamatsu, T. Kobayashi, The human RNA polymerase I transcription terminator complex acts as a replication fork barrier that coordinates the progress of replication with rRNA transcription activity. *Mol. Cell. Biol.* **35**, 1871–1881 (2015).

23. M. D. Burkhalter, J. M. Sogo, rDNA enhancer affects replication initiation and mitotic recombination. *Mol. Cell* **15**, 409–421 (2004).

24. R. Ranjan, J. Snedeker, M. Wooten, C. Chu, S. Bracero, T. Mouton, X. Chen, Differential condensation of sister chromatids acts with Cdc6 to ensure asynchronous S-phase entry in *Drosophila* male germline stem cell lineage. *Dev. Cell* **57**, 1102–1118.e7 (2022).

25. V. Tran, C. Lim, J. Xie, X. Chen, Asymmetric division of *Drosophila* male germline stem cell shows asymmetric histone distribution. *Science* **338**, 679–682 (2012).

26. M. Wooten, J. Snedeker, Z. F. Nizami, X. Yang, R. Ranjan, E. Urban, J. M. Kim, J. Gall, J. Xiao, X. Chen, Asymmetric histone inheritance via strand-specific incorporation and biased replication fork movement. *Nat. Struct. Mol. Biol.* **26**, 732–743 (2019).

27. J. Xie, M. Wooten, V. Tran, B.-C. Chen, C. Pozmanter, C. Simbolon, E. Betzig, X. Chen, Histone H_3 threonine phosphorylation regulates asymmetric histone inheritance in the *Drosophila* male germline. *Cell* **163**, 920–933 (2015).

28. M. Van Doren, A. L. Williamson, R. Lehmann, Regulation of zygotic gene expression in *Drosophila* primordial germ cells. *Curr. Biol.* **8**, 243–246 (1998).

29. M. P. Zeidler, N. Perrimon, D. I. Strutt, Polarity determination in the *Drosophila* eye: A novel role for unpaired and JAK/STAT signaling. *Genes Dev.* **13**, 1342–1353 (1999).

30. S. E. McGuire, P. T. Le, A. J. Osborn, K. Matsumoto, R. L. Davis, Spatiotemporal rescue of memory dysfunction in *Drosophila*. *Science* **302**, 1765–1768 (2003).

31. M. Inaba, M. Buszczak, Y. M. Yamashita, Nanotubes mediate niche-stem-cell signalling in the *Drosophila* testis. *Nature* **523**, 329–332 (2015).

32. D. Tautz, C. Tautz, D. Webb, G. A. Dover, Evolutionary divergence of promoters and spacers in the rDNA family of four *Drosophila* species. Implications for molecular coevolution in multigene families. *J. Mol. Biol.* **195**, 525–542 (1987).

33. M. Zaccai, H. D. Lipshitz, Differential distributions of two adducin-like protein isoforms in the *Drosophila* ovary and early embryo. *Zygote* **4**, 159–166 (1996).

34. B. Riggleman, P. Schedl, E. Wieschaus, Spatial expression of the *Drosophila* segment polarity gene armadillo is posttranscriptionally regulated by wingless. *Cell* **63**, 549–560 (1990).

35. M. Jagannathan, R. Cummings, Y. M. Yamashita, A conserved function for pericentromeric satellite DNA. *eLife* **7**, e34122 (2018).

36. A. M. Huang, E. J. Rehm, G. M. Rubin, Quick preparation of genomic DNA from *Drosophila*. *Cold Spring Harb. Protoc.* **2009**, pdb prot5198 (2009).

37. J. O. Nelson, A. Slicko, Y. M. Yamashita, The retrotransposon R2 maintains *Drosophila* ribosomal DNA repeats. bioRxiv 2021.2007.2012.451825 [**Preprint**]. 12 July 2021. https://doi.org/10.1101/2021.07.12.451825.

38. A. V. Emelyanov, E. Vershilova, M. A. Ignatyeva, D. K. Pokrovsky, X. Lu, A. Y. Konev, D. V. Fyodorov, Identification and characterization of ToRC, a novel ISWI-containing ATP-dependent chromatin assembly complex. *Genes Dev.* **26**, 603–614 (2012).

39. J. Lu, S. J. Marygold, W. H. Gharib, B. Suter, The aminoacyl-tRNA synthetases of *Drosophila melanogaster*. *Fly (Austin)* **9**, 53–61 (2015).

40. R. T. Cox, A. C. Spradling, Clueless, a conserved *Drosophila* gene required for mitochondrial subcellular localization, interacts genetically with parkin. *Dis. Model. Mech.* **2**, 490–499 (2009).

41. M. J. Matunis, E. L. Matunis, G. Dreyfuss, Isolation of hnRNP complexes from *Drosophila melanogaster*. *J. Cell Biol.* **116**, 245–255 (1992).

42. S. C. Renn, B. Tomkinson, P. H. Taghert, Characterization and cloning of tripeptidyl peptidase II from the fruit fly, *Drosophila melanogaster*. *J. Biol. Chem.* **273**, 19173–19182 (1998).

43. S. J. Lee, R. Feldman, P. H. O'Farrell, An RNA interference screen identifies a novel regulator of target of rapamycin that mediates hypoxia suppression of translation in *Drosophila* S2 cells. *Mol. Biol. Cell* **19**, 4051–4061 (2008).

44. L. Dorstyn, K. Mills, Y. Lazebnik, S. Kumar, The two cytochrome c species, DC3 and DC4, are not required for caspase activation and apoptosis in *Drosophila* cells. *J. Cell Biol.* **167**, 405–410 (2004).

45. K. N. Rugjee, S. R. Chaudhury, K. al-Jubran, P. Ramanathan, T. Matina, J. Wen, S. Brogna, Fluorescent protein tagging confirms the presence of ribosomal proteins at *Drosophila* polytene chromosomes. *PeerJ* **1**, e15 (2013).

46. K. Takata, H. Yoshida, F. Hirose, M. Yamaguchi, M. Kai, M. Oshige, I. Sakimoto, O. Koiwai, K. Sakaguchi, *Drosophila* mitochondrial transcription factor A: Characterization of its cDNA and expression pattern during development. *Biochem. Biophys. Res. Commun.* **287**, 474–483 (2001).

47. M. Jagannathan, N. Warsinger-Pepe, G. J. Watase, Y. M. Yamashita, Comparative analysis of satellite DNA in the *Drosophila melanogaster* species complex. *G3 (Bethesda)* **7**, 693–704 (2017).

48. Q. Zhang, N. A. Shalaby, M. Buszczak, Changes in rRNA transcription influence proliferation and cell fate within a stem cell lineage. *Science* **343**, 298–301 (2014).
